# Dietary DHA Regulated the Androgen Production in Male Chinese Tongue Sole *Cynoglossus semilaevis*

**DOI:** 10.1155/anu/9318358

**Published:** 2025-01-25

**Authors:** Jiahao Liu, Qiang Ma, Feiran Zhang, Qingyan Gao, Zhijun Zhang, Yuliang Wei, Mengqing Liang, Houguo Xu

**Affiliations:** ^1^State Key Laboratory of Mariculture Biobreeding and Sustainable Goods, Yellow Sea Fisheries Research Institute, Chinese Academy of Fishery Sciences, 106 Nanjing Road, Qingdao 266071, China; ^2^Laboratory for Marine Fisheries Science and Food Production Processes, Qingdao Marine Science and Technology Center, 168 Wenhai Road, Qingdao 266237, China

**Keywords:** aquaculture industry, fish reproduction, food nutrition, marine flatfish, nutritional regulation, testicular genes

## Abstract

Long-chain polyunsaturated fatty acids (LC-PUFA) play key roles in sex steroid hormone synthesis in fish. Regarding docosahexaenoic acid (DHA), currently the regulating effects were mostly evaluated in female fish rather than males. This study aimed to investigate the DHA effects on the sex steroid hormone production in male Chinese tongue sole. Three experimental diets were prepared, containing different DHA levels: a control (C) group (5.16% DHA of total fatty acids [TFA]), a low DHA group (DHA-L, 8.93%), and a high DHA group (DHA-H, 16.47%). A 58-day feeding experiment was conducted, and each diet was fed to triplicate tanks of fish. Additionally, an in vitro study with Leydig's cells of this species was conducted to validate the in vivo results. The concentration of testosterone (T) and 11-ketotestosterone (11-KT) in the serum increased with increasing levels of dietary DHA. Dietary DHA significantly upregulated the expression of steroid hormone biosynthetic genes *p450c17*, *hsd17b1*, *hsd3b1*, *aromatase*, *hsd11b2*, and *p45011b* in the testis. The protein expression of Hsd17b1 in the testis of the DHA-H group was significantly higher compared to the other two groups, while the expression level of P450c17 showed an increasing trend with increasing dietary DHA levels. However, the in vitro results confirmed that the final concentration of DHA at 50 μmol/L could significantly increase the gene expression of *p450c17* in Leydig's cells. In conclusion, dietary DHA may promote the synthesis of T and 11-KT through the regulation of protein (Hsd17b1 and P450c17) and gene (*p450c17*, *hsd17b1*, *hsd3b1*, *aromatase*, *hsd11b2*, and *p45011b*) expression of a series of key steroid hormone biosynthetic enzymes in male Chinese tongue sole.

## 1. Introduction

Long-chain polyunsaturated fatty acids (LC-PUFA) are essential fatty acids for some marine fish species. The LC-PUFA play key roles in a series of physiological processes [1–3]. In particular, for fish reproduction, some LC-PUFA, such as docosahexaenoic acid (DHA) and arachidonic acid (ARA), largely influence the spawning capacity, hatching rate, fertilization rate, and offspring quality of fish [4–8]. In fish species such as rainbow trout *Oncorhynchus mykiss*, cobia *Rachycentron canadum*, silver pomfret *Pampus argenteus*, and Chinese tongue sole *Cynoglossus semilaevis*, the reproduction-regulating effects of DHA have been widely observed [9–14]. However, most of these studies focused on n-3 LC-PUFA enriched oil or fish oil [1, 4, 15]. Due to the coexistence of EPA and DHA [15], it was difficult to accurately assess the reproduction-regulating effects of individual DHA. Less studies used pure DHA in the experimental diets.

Besides, most of the previous relevant studies involved both female and male fish, as well as the offspring performance [1, 4, 9]. Few studies have investigated the sexual dimorphic effects of DHA on fish reproduction [16]. For male fish, DHA is important to sperm production, sperm motility and fertilization ability [6, 17]. In a study on European eel *Anguilla japonica*, DHA in the testis was found to be significantly correlated with serum testosterone (T) and 11-ketotestosterone (11-KT) concentration [18]. The promotion of T synthesis by DHA has also been observed in humans [19] and rats [20]. All these studies suggest that DHA plays a significant role in male reproduction. To date, still little information is available about the DHA function in the reproductive physiology of male fish. In a marine carnivorous fish species, our previous research has shown that the DHA:EPA ratio differentially regulated the sex steroid hormone synthesis in mature female and male tongue sole [16]. Males seemed to require more DHA but less EPA for gonadal steroidogenesis than females.

Chinese tongue sole *C. semilaevis* is a valuable mariculture fish species in Northern China. This species has a unique characteristic of sexual dimorphism. The male adult Chinese tongue sole usually weighed only 1/4 to 1/2 that of female fish [21, 22]. The male and female gonads of this species usually do not develop simultaneously. The degraded germplasm resources of male Chinese tongue sole have been a threat to the successful breeding activities, leading to poor fertility and low larval survival [23]. Nutritional manipulation of the reproductive performance of male fish could be beneficial to the improvement of breeding success of this species. In this study, targeting at the steroid biosynthesis, which is a primary process in reproduction, we formulated diets with different DHA levels and assessed the effects of dietary DHA on androgen biosynthesis in male Chinese tongue sole. The involved mechanisms, mostly regarding the effects on gene and protein expression of androgen biosynthetic enzymes, were also investigated, along with an in vitro validation study.

## 2. Materials and Methods

### 2.1. Experimental Diets

Three isonitrogenous and isocaloric experimental diets (crude protein 51.5% and crude lipid 10.5%) were formulated, containing different levels of DHA-enriched oil (52% DHA, in the form of triglycerides): 0.10%, 0.95%, and 2.65% (Table 1). The DHA content in the three experimental diets accounted for 5.16%, 8.93%, and 16.47% of the total fatty acids (TFA, Table 2), respectively, and were designated as control (C), low DHA group (DHA-L), and high DHA group (DHA-H), respectively. All ingredients were sieved through a 60-mesh sieve, accurately weighed, and mixed thoroughly. Subsequently, the pellets were made using a pelleting machine and dried at 56°C. The dried diets were preserved at 4°C before the feeding trial [24].

### 2.2. Experimental Fish and Feeding Management

The same batch of experimental juvenile Chinese tongue sole (5 months old, average initial body weight, 10.46 ± 0.15 g) with no dark color on the abdominal side were selected for the experiment. The juveniles were in the gonadal development stage of II. The fish were purchased from Chengtai Aquaculture Co., Ltd. (Rizhao, China). The experimental fish were transported to Haiyang Aquaculture Co., Ltd. (Haiyang, China), where the feeding experiment was conducted. First, fish were fed a commercial feed for 30 days to acclimate to the experiment conditions. Subsequently, the experimental fish were fasted for 24 h before randomly distributed into nine polyethylene tanks (72 cm × 72 cm × 56 cm), with 15 fish per tank and three replicates per group. The fish were fed to satiation twice daily (7:30 and 19:00). Residual feed on the tank bottom was cleaned daily, and the tank walls were cleaned every 5 days. The experimental water was sourced from both seawater and shallow groundwater and mixed in a reservoir before use. The water temperature ranged from 19 to 28°C; salinity, 28–30; and dissolved oxygen greater than 6.08 mg/L during the experimental period.

After 58 days of feeding, the experimental fish were fasted for 24 h before sampling. The number and weight of fish in each tank were recorded, and 12 fish were selected from each tank. After anesthetized with MS-222, the weight of each fish was measured, and the lateral fin samples were collected. Blood was collected from the caudal vein, which was allowed to clot for 2 h at room temperature and then for another 6 h at 4°C. Following centrifugation at 4000 × *g* for 10 min, the serum was collected and stored at −80°C. Subsequently, the brain, liver, gonad, muscle, and skin on the eyeless side (both skin samples with and without dark pigment) of each fish were harvested and placed in cryopreservation tubes, which were then stored in liquid nitrogen and subsequently transferred to a −80°C freezer for future use. All handling and sampling practices in this study were reviewed and approved by the Animal Care and Use Committee of Yellow Sea Fisheries Research Institute (protocol code ACUC202208111518).

### 2.3. Proximate and Fatty Acid Compositions of Fish Tissues

The proximate composition in the experimental diets was determined according to the AOAC methods. The moisture content in the liver and muscle was measured by freeze-drying for 24 h with a freeze dryer (FDU-1100, Tokyo Rikakikai, Co. Ltd., Tokyo, Japan). The crude lipid content was determined via Soxhlet extraction (FOSS Soxtec 2050, Hillerod, Denmark) using petroleum ether as the solvent. The crude protein content was measured using the Kjeldahl method with a Kjeldahl analyzer (FOSS 2300, Hillerod, Denmark). The ash content was assayed with incineration at 550°C for 8 h.

The fatty acid compositions of diets, liver, and muscle were assayed by the gas chromatography method (GC-2010 pro, Shimadzu, Tokyo, Japan). The fatty acids in lyophilized samples were esterified with KOH-methanol and BF_3_ successively in a 75°C water bath. Fatty acid methyl esters were extracted with hexane and then subjected to the gas chromatography, which was equipped with a flame ionization detector and a fuzed silica capillary column (SH-RT-2560, 100 m × 0.25 mm × 0.20 μm). The column temperature increase was programed: from 100°C up to 190°C at a rate of 10°C/min; then from 190°C–200°C at a rate of 0.3°C/min; and then from 200 to 230°C at a rate of 4°C/min. Both injector and detector temperatures were 230°C. The fatty acid contents were expressed as %TFA. The detailed program was similar to Liu et al. [25].

### 2.4. Sex Identification of Experimental Fish

Briefly, the DNA was extracted from the fin using a Marine Animal Tissue Genomic DNA Extraction Kit (TIANGEN BIOTECH, Beijing, China). After extraction, the DNA was subjected to PCR amplification with sex-specific markers (F: CCTAAATGATGGATGTAGATTCTGTC, R: GATCCAGAGAAAATAAACCCAGG). The PCR products were then electrophoresed on a 2% agarose gel at 180 V for 30 min. At the end of the electrophoresis, the bands were observed under UV conditions. A single band (169 bp) indicated genetic male fish, while two bands (169 bp/134 bp) indicated genetic female fish. The genetic female Chinese tongue sole may include both female and pseudomale fish, but all Chinese tongue sole with one band can be considered male fish. In this study, male fish were selected for subsequent experiments. More details on sex identification of Chinese tongue sole can be found in a previous publication of Liu et al. [25].

### 2.5. The Analysis of Androgen in Serum

The measurement of T and 11-KT in serum was performed using commercial ELISA kits (T: #H090-1-1; 11-KT: #H088) provided by Nanjing Jiancheng Bioengineer Institute (Nanjing, China). Briefly, the standard curve was plotted following the procedures stipulated in the instruction manual using the standards provided in the kit. Subsequently, the serum was processed in accordance with the kit's instructions. After that, the absorbance is measured with a microplate reader (TECAN Infinite M200, Switzerland), and the obtained values are put into the standard curve to calculate the final concentration.

### 2.6. Quantitative Real-Time Polymerase Chain Reaction (qRT-PCR)

The total RNA in the gonad was extracted using RNA iso Plus (TaKaRa Biotechnology Co., Ltd., Dalian, China). Subsequently, reverse transcription was carried out with the Evo M-MLVRT Mix kit with gDNA Clean for qPCR (provided by Accurate Biotechnology Co., Ltd., China) in accordance with the manufacturer's instructions.

Based on the sequences from GenBank, specific primers for the target gene and reference genes were designed and synthesized by Qingke Biotechnology Co., Ltd. (Qingdao, China) (Table 3). In this experiment, *β*-actin and *β*2M of Chinese tongue sole were used as the reference genes. The primer validation steps, reaction system, and PCR program were according to Xiong et al. [26]. The mRNA expression level of the target gene was calculated using the 2^−*ΔΔCt*^ method.

### 2.7. Western Blot (WB) Analysis

Briefly, the protein was extracted from the testis tissue of the Chinese tongue sole using RIPA lysis buffer, and the protein concentration of the samples was precisely quantified using a BCA kit (#P0010S, Beyotime, China). SDS–PAGE gel electrophoresis was then employed to separate the protein in the samples, and the protein was transferred onto a polyvinylidene fluoride (PVDF) membrane. After blocking, washing, and incubation with the target protein antibody, as well as incubation with the corresponding secondary antibody (Table 4), visualization was achieved using BeyoECL Star (super-sensitive ECL chemiluminescent kit, #P0018AS, Beyotime, China) from Beyotime. Exposure and photography were carried out using the Tanon 5200 series automatic chemiluminescence image analyzer (Tianneng Life Science Co., Ltd. Shanghai, China), and quantitation was performed using Image J.

### 2.8. The In Vitro DHA Incubation Study

The Chinese tongue sole testis cell was provided by the laboratory of Songlin Chen in Yellow Sea Fisheries Research Institute, Chinese Academy of Fishery Sciences. The complete medium consisted of 20% fetal bovine serum (#A5669701, Gibco, China), 2% triple antibiotics (penicillin-streptomycin-amphotericin B, #C0223, Beyotime, China), 5 ng/mL growth factors, including bFGF, EGF, and LIF (#P5453, #P6114, #P5354, Beyotime, China), and 27.5 μmoL/L mercaptoethanol (#GUSA-R001, Haixing Biotechnology Co., Ltd., China). L-15 (#11415064, Gibco, China) was used as the basic medium. Once the cells grew to cover the bottom of the T25 flask (#FFLK075, Beyotime, China), the medium was discarded, and the flask was washed twice with DPBS. Then, 3 mL of 0.25% trypsin (#T1300, Solarbio, China) was added for digestion for about 10 s. Two milliliters of trypsin were aspirated, and the digestion was allowed to continue for ~5 min. After the cells were detached, the complete medium containing serum was added to neutralize the trypsin. The cell suspension was evenly distributed into a 6-well plate, and the wells were filled with the complete medium to 2 mL each. DHA (#D2534, Sigma-Aldrich, USA) was added to the complete medium using 2% bovine serum albumin (BSA) (#A8020, Solarbio, China) as the solvent. The final DHA concentrations were 10, 50, and 100 umol/L, respectively. The C group received the same volume of 2% BSA. After incubation for 24 h, the medium was discarded, and the cells were washed twice with PBS. RNA lysis buffer was then added, and the cells were left to sit on ice for 5 min. Subsequently, the total RNA of cells was extracted for subsequent q-PCR experiments.

### 2.9. Statistical Methods

All data were analyzed using one-way ANOVA in SPSS 16.0 (SPSS Inc. Chicago, USA). After passing the test of homogeneity of variances, multiple comparisons were conducted using Tukey's test. The calculated results in the tables and figures are presented as mean ± standard error. Significant differences were considered when *p* < 0.05.

## 3. Results

### 3.1. Genetic Sex Determination

**Figure 1:**
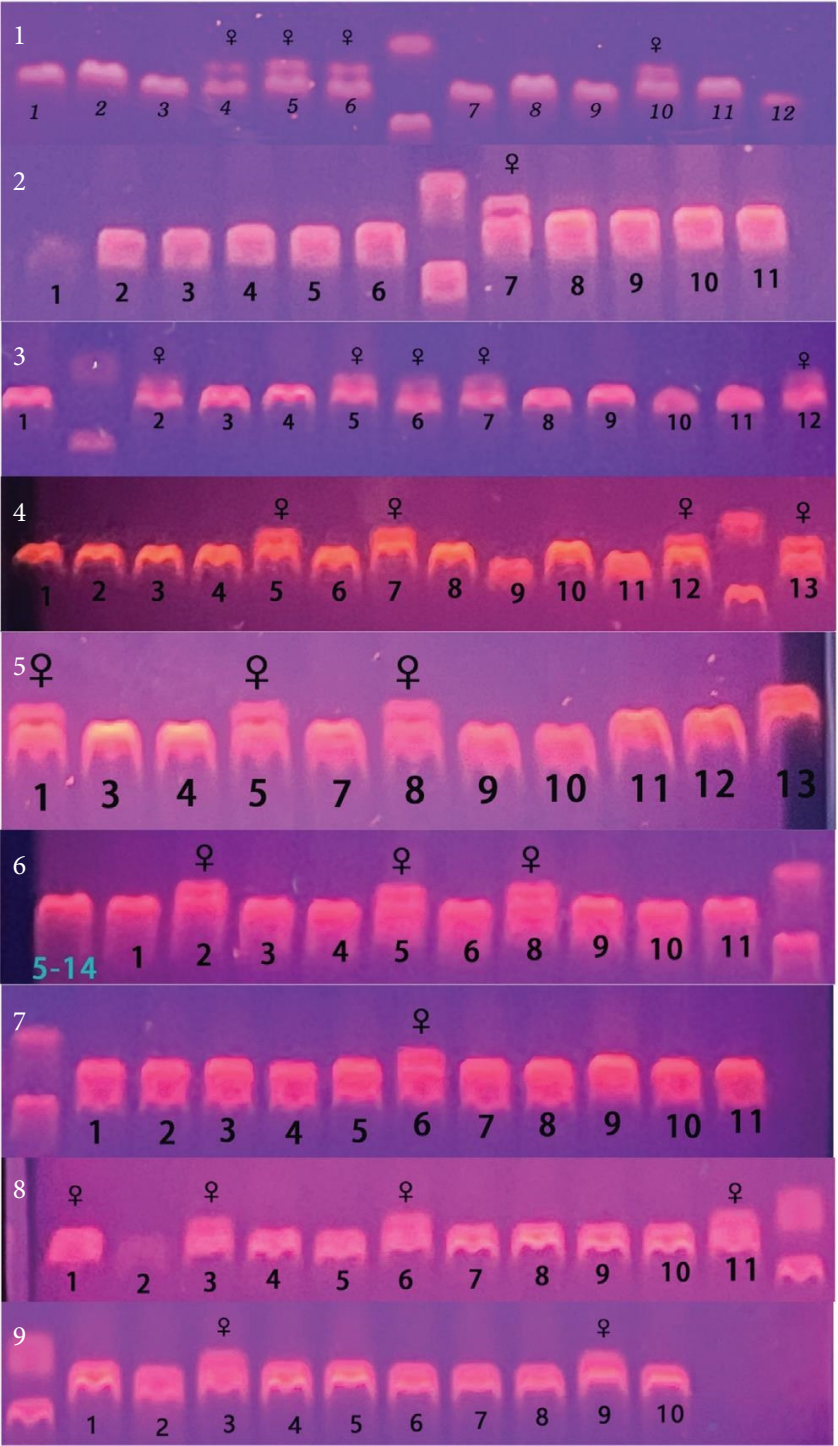
PCR validation of genetic sex of Chinese tongue sole. Group C included 1, 2, and 3; Group DHA-L included 4, 5, and 6; Group DHA-H included 7, 8, and 9. C, control; DHA-H, high docosahexaenoic acid group; DHA-L, low docosahexaenoic acid group; PCR, polymerase chain reaction.

The total number of genetic female fish in group C, DHA-L, and DHA-H was 10, 10, and 7, respectively, and that of male fish in the three groups was 25, 28, and 25, respectively (Figure 1; Table 5).

### 3.2. Growth Performance and Proximate Composition of Fish Tissue

**Figure 2:**
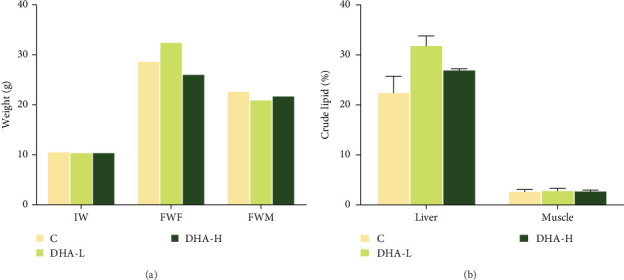
The weight information of experiment fish (A) and the crude lipid content (B) of muscle and liver in male fish at the end of the feeding experiment. The crude lipid contents of liver and muscle are calculated based on wet weight. C, control; DHA, docosahexaenoic acid; DHA-H, high docosahexaenoic acid group; DHA-L, low docosahexaenoic acid group; FWF, final mean weight of females (genetic); FWM, final mean weight of males; IW, initial mean weight.

All experimental fish initially weighed about 10 g. At the end of the feeding experiment, the weight difference between the female and male fish in the same treatment group had begun, and the weight of genetically female fish was generally larger than the male fish (Figure 2). Dietary DHA content had no significant effects on the body weight of the male and female fish (*p* > 0.05) and had no significant effects on total lipid content in muscle and liver of male fish (*p* > 0.05).

### 3.3. Fatty Acid Composition in Tissues of Male Fish

The liver DHA content in the DHA-H group was significantly (*p* < 0.05) higher than that in the C and DHA-L groups, while no significant (*p* > 0.05) difference was observed between the latter two groups (Table 6). In contrast, the liver 18:1n-9 content in the DHA-H group was significantly (*p* < 0.05) lower than that in the C and DHA-L groups. Very similar results were observed in the muscle.

### 3.4. Concentrations of T and 11-KT in Serum

**Figure 3:**
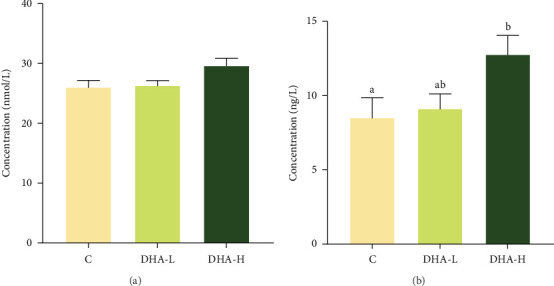
Effects of dietary DHA on the androgen ((A) testosterone and (B) 11-ketotestosterone) concentration in serum of male Chinese tongue sole. For a specific androgen, data bars not sharing the same letter are significantly different (*p* <  0.05). C, control; DHA, docosahexaenoic acid; DHA-H, high docosahexaenoic acid group; DHA-L, low docosahexaenoic acid group.

The serum T content exhibited an increasing trend with increasing dietary DHA levels, but no significant (*p* > 0.05) difference was observed among groups (Figure 3). The content of 11-KT increased significantly (*p* < 0.05) with the increasing DHA contents in the diet, and the DHA-H group showed a significantly higher level than the C group.

### 3.5. Expression of Steroid Hormone Biosynthetic Genes in the Testis

**Figure 4:**
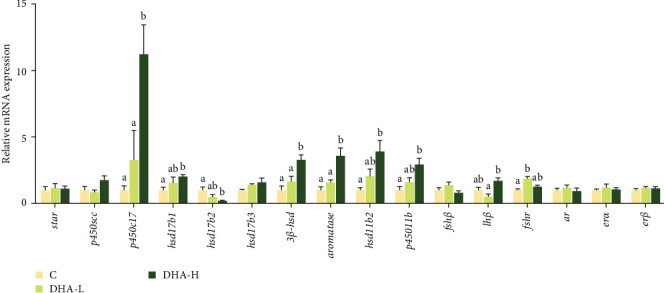
Effects of dietary DHA on the expression of steroid hormone biosynthetic genes in the testis of male Chinese tongue sole. For a specific gene, data bars not sharing the same letter are significantly different (*p* <  0.05). The expression of each gene is normalized according to the DHA-L group. C, control; DHA, docosahexaenoic acid; DHA-H, high docosahexaenoic acid group; DHA-L, low docosahexaenoic acid group.

The expression levels of *p450c17*, *hsd17b1*, *hsd3b1*, *hsd11b2*, *p45011b*, and *aromatase* were significantly (*p* < 0.05) upregulated by increasing DHA levels in the diet (Figure 4). The expression of *hsd17b3* showed a similar changing trend, but no significant difference was observed among groups (*p* > 0.05).

### 3.6. Gene Expression of Gonadotropin-Releasing Hormone (GnRH) and Its Receptor (GnRHR), As well As Those Associated With the PKC/MAPK Pathway in the Brain of Male Fish

**Figure 5:**
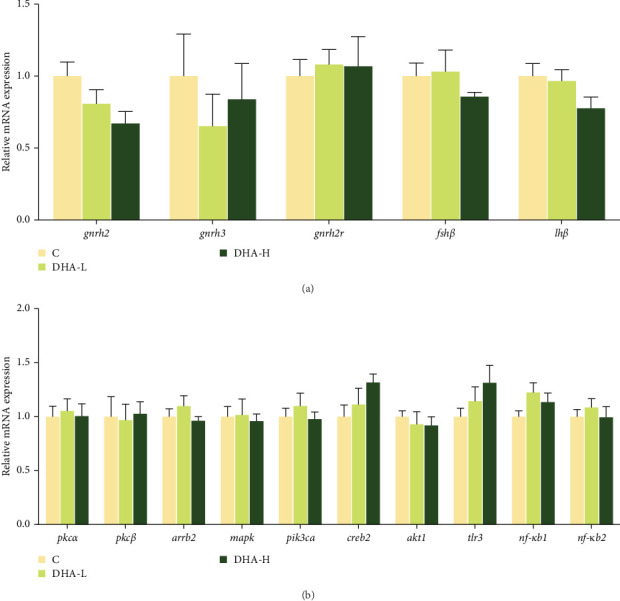
Effects of dietary of DHA on gene expression of gonadotropin-releasing hormone (*gnrh*) and its receptor (A), as well as those of the PKC/MAPK pathway genes (B) in the brain of male Chinese tongue sole. The expression of each gene is normalized according to the DHA-L group. C, control; DHA, docosahexaenoic acid; DHA-H, high docosahexaenoic acid group; DHA-L, low docosahexaenoic acid group.

The expression of *gnrh2* showed decreasing trends with increasing DHA levels in the diet, but no significant (*p* > 0.05) differences were observed among groups (Figure 5). There were also no significant differences in the expression of other genes studied (*p* > 0.05).

### 3.7. The Protein Expressions of P450c17 and Hsd17b1

**Figure 6:**
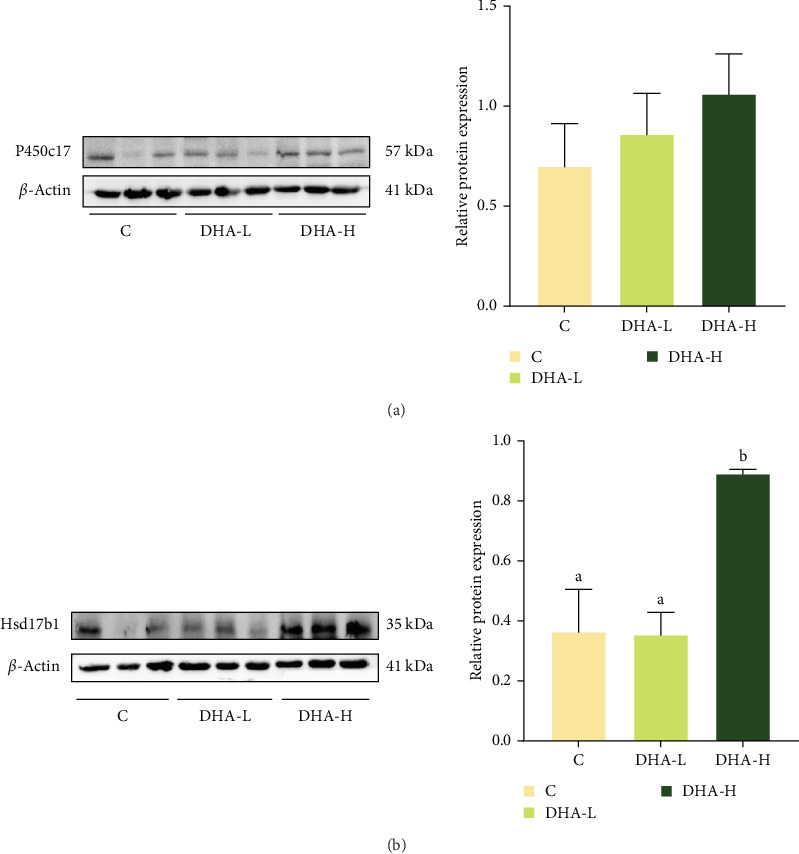
Effects of dietary DHA on the protein expression of P450c17 (A) and Hsd17b1 (B) in the testis of male Chinese tongue sole. For a specific protein, data bars not sharing the same letter are significantly different (*p* <  0.05). C, control; DHA, docosahexaenoic acid; DHA-H, high docosahexaenoic acid group; DHA-L, low docosahexaenoic acid group.

The protein expression of P450c17 showed an increasing trend with increasing DHA levels in the diet, although no significant difference (*p* > 0.05) was observed among groups (Figure 6). The DHA-H group showed significantly (*p* < 0.05) higher protein expression of Hsd17b1 than the other two groups.

### 3.8. Expression of Steroid Hormone Biosynthetic Genes in the In Vitro Study

**Figure 7:**
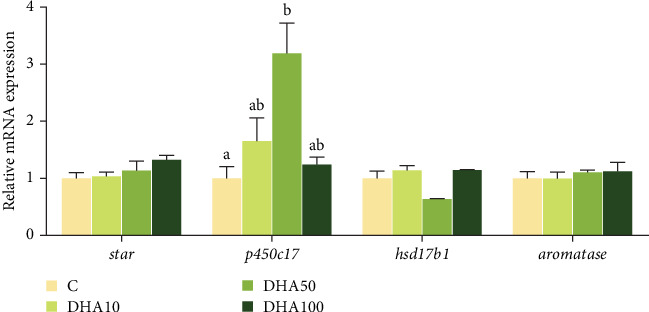
Effects of different DHA concentrations on steroid hormone biosynthetic genes in the in vitro study. For a specific gene, data bars not sharing the same letter are significantly different (*p* <  0.05). The expression of each gene is normalized according to the C group. C, control; DHA, docosahexaenoic acid; DHA-H, high docosahexaenoic acid group; DHA-L, low docosahexaenoic acid group.

The expression level of *p450c17* increased with the increase of DHA concentration up to 50 μmol/L but decreased thereafter (Figure 7). The *p450c17* expression level in the group with 50 μmol/L DHA was significantly (*p* < 0.05) higher compared to the C group. The expression of *hsd17b1* in the group with 50 μmol/L DHA was significantly (*p* < 0.05) lower compared to the groups with 10 or 100 μmol/L DHA. There were no significant differences (*p* > 0.05) in the gene expression of *star* and *aromaste* among experimental groups.

## 4. Discussion

The effects of DHA on fish reproduction have been reported in a variety of fish species [7, 12, 27]. In our previous studies [28], it was found that DHA significantly contributed to the sex difference of fatty acids in all tissues of Chinese tongue sole, especially in the gonads. The content of DHA in ovaries is significantly lower than that in testes, which may indicate that the testes need more DHA than ovaries. Based on these previous findings, this study evaluated the influence of DHA on male Chinese tongue sole, focusing on steroidogenesis.

**Figure 8:**
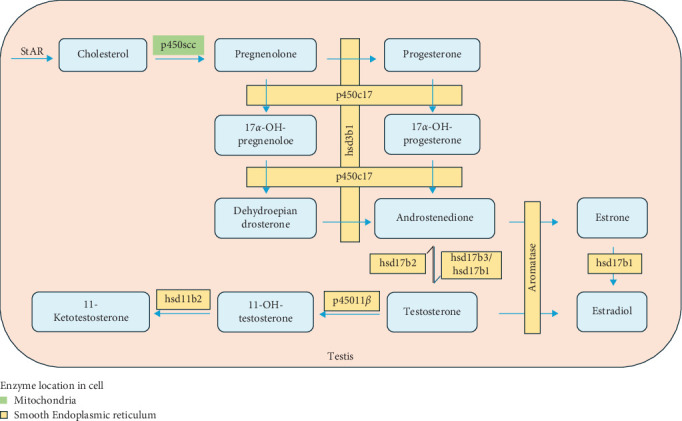
Sex steroid synthesis pathway in teleosts (source data from [29–31]).

In this study, high levels of DHA in the diet promoted the synthesis and secretion of androgen (T and 11-KT) levels in male fish. This result was well consistent with and explained by the regulation of androgen biosynthetic genes by dietary DHA. The present results also indicate that *p450c17* could be a main target gene of the DHA influence (Figure 8), which was further validated by the in vitro experiment.

*P450c17* is an enzyme with biofunctions at one active site, namely 17*α*-hydroxylase activity and 17,20-lyase activity [32, 33] (Figure 8). The 17*α*-hydroxypregnenolone and 17*α*-hydroxyprogesterone were synthesized by 17*α*-hydroxylation of pregnenolone and progesterone, respectively. Dehydroepiandrosterone and androstenedione are then synthesized through the 17,20-lyase activity of this enzyme [34]. Therefore, P450c17 is a key enzyme in the sex steroid synthesis pathway. In a previous study published by our research group, it was found that the *p450c17* gene expression in the testis of Chinese tongue sole was upregulated by a higher dietary DHA/EPA ratio [11]. Nevertheless, in this study, the protein expression of *P450c17* was not significantly regulated by dietary DHA, although there was an obvious elevation with increasing dietary DHA levels. P450c17 is a phosphoprotein, and in human studies, it was found that 17,20 lyase preferentially catalyzed the conversion of 17*α*-hydroxypregnenolone to dehydroepiandrosterone, while the efficiency of catalytic conversion of 17*α*-hydroxypregnenolone to androstenedione was very low [35]. Phosphorylation of P450c17 on serine and threonine residues selectively increases the 17,20 lyase activity. This may potentially affect the expression level of the P450c17 protein.

Another target gene of DHA regulation could be *hsd17b*. The 17*β* hydroxysteroid dehydrogenase (17*β*-Hsd) is an important enzyme in the conversion of androstenedione to T (Figure 8). There are as many as 15 subtypes of *hsd17b* [36]. In this study, the gene expression of three subtypes (*hsd17b1*, *hsd17b2*, and *hsd17b3*) was mainly studied. According to previous reports, *hsd17b3* is generally considered to be a key enzyme that catalyzes the conversion of androstenedione to T and is essential for T synthesis [37, 38]. In the currently available fish research on fish species, it has been shown that Hsd17b3 had higher expression levels in the testis compared to the ovary, with even more pronounced differences across different stages of testicular development. In Nile tilapia *Oreochromis niloticus*, it had been observed that the *hsd17b3* was exclusively expressed in the testis [39]. In both pipefish *Syngnathus scovelli* and olive flounder *Paralichthys olivaceus*, the expression of *hsd17b3 in the* testes was upregulated, and during the developmental stages I, II, and IV of the olive flounder testes, the specific enzyme activity of Hsd17b3 was higher compared to the ovary [40, 41]. These findings indicate a more significant role of this enzyme in the androgen synthesis. In this study, the *hsd17b3* gene expression tended to increase with increasing dietary DHA levels, which was very similar to the T changes. The result appeared to further substantiate the pivotal role of this enzyme in the androgen synthesis. In contrast, *hsd17b2* mainly converts high-activity hormones to low-activity ones, such as catalyzing the conversion of T to androstenedione and the conversion of estradiol (E_2_) to estrone [42]. This may be the reason why the expression of *hsd17b2* in this study decreased with the addition of DHA. The expression levels of Hsd17b1 gene and protein increased significantly with the addition of DHA. It has been reported that this subtype usually has a high affinity with estrone and is involved in the conversion of estrone to E_2_ [43], which have been used as indicators of positive regulation of T in mice and Chinese tongue sole [11, 44].

The 11-KT is considered to be the most important androgen in fish spermatogenesis and plays an important role in sex determination, stronger than T [45]. 11-KT is produced using T as substrate, catalyzed by 11*β*-hydroxylase (*p45011β*) and hydroxyl steroid 11-*β* dehydrogenase 2 (*hsd11b2*). In this study, the elevation of 11-KT level in serum by DHA was consistent with the up-regulation of *p45011β* and *hsd11β2* expression in the testis.

In this study, dietary DHA did not promote the expression of GnRH gene (*gnrh2*) in the brain of male fish, although there was a down-regulation trend. Dietary DHA also had no significant effect on genes in the PKC/MAPK pathway. This was not consistent with the results of Tran et al. [46], which showed that in the neuronal cell model mHypoA-GnRH/GFP, DHA stimulated the production of GnRH by mediating the GPR120 receptor on the cell membrane to downstream PKC/MAPK and PI3K signal transduction. The effects of PUFA on GnRH and gonadotropin have been reported in other studies. In a study on Chinese tongue sole, it was found that ARA in the diet also promoted the GnRH production in the brain, and the regulation of PKC/MAPK pathway was involved [47]. In rat primary cells and L*β*T2 gonadotropin cell lines, linoleate inhibited the GnRHa production by increasing luteinizing hormone (LH) levels and LH*β* mRNA expression, which could be due to the activation of two different kinases PKC and ERK1/2 in gonadotropin cells [48]. They may phosphorylate several elements of GnRHR signaling, resulting in desensitization. There was a lack of information on the precise targets of unsaturated fatty acids in the GnRH production pathway.

In the in vitro study, the expression of *p450c17* gene was the highest when the DHA concentration was 50 μmol/L. Other key genes of steroid synthesis had too low Cq values to be detected. This may be related to the absence of gonadotropin in the in vitro experiment. The hypothalamic–pituitary–gonadal axis plays an important role in regulating fish reproduction. The brain releases GnRH to act on the pituitary gland, which receives the signal to secrete follicle-stimulating hormone (FSH) and LH, and FSH and LH stimulate the gonads to promote the synthesis of sex steroid hormones. In European bass [49], Senegalese sole [50], and Japanese eel [37], human chorionic gonadotropin promoted the fatty acid metabolism and steroid hormone synthesis. Therefore, the role of gonadotropin should be taken into account in future in vitro experiments.

## 5. Conclusion

Dietary addition of DHA had a positive effect on the synthesis of sex steroid hormone in male Chinese tongue sole. Dietary DHA promoted the gonadal biosynthesis of T and 11-KT, which could be via the regulation of *p450c17*, *hsd17b1*, *hsd3b1*, *hsd11b2*, and *p45011b*. The DHA effects could be at the transcription or translation level, depending on specific genes. This study enhances our knowledge on the DHA function in sex steroid synthesis in fish, providing insights for how to enhance the reproductive performances of male fish. Further studies are needed to elucidate the mechanisms involved in the effects of dietary DHA on specific steroidogenic proteins.

## Figures and Tables

**Table 1:** Formulation and proximate composition of the experimental diets used in this study (% dry matter).

Ingredients	C	DHA-L	DHA-H
Fish meal	40	40	40
Soybean meal	22	22	22
Wheat gluten	9	9	9
Wheat meal	16	16	16
Vitamin premix^1^	1	1	1
Mineral premix^2^	0.5	0.5	0.5
L-ascorbyl-2-polyphosphate	0.2	0.2	0.2
Choline chloride	1	1	1
Monocalcium phosphate	1	1	1
Ethoxyquinoline	0.05	0.05	0.05
Calcium propionate	0.1	0.1	0.1
Betaine	0.3	0.3	0.3
Soya lecithin	1	1	1
Soybean oil	3	3	3
DHA-enriched oil^3^	0.10	0.95	2.65
EPA-enriched oil^3^	1.03	0.93	0.71
ARA-enriched oil^3^	0.7	0.7	0.7
Olive oil	3.02	2.27	0.79
Total	100	100	100
Proximate composition	—	—	—
Moisture	4.18	4.84	4.66
Crude protein	51.56	51.64	51.34
Crude lipid	10.78	10.75	10.99
Ash	10.62	10.44	10.45

Abbreviations: ARA, arachidonic acid; C, control; DHA, docosahexaenoic acid.

^1^Vitamin premix (IU or g/kg premix): vitamin A acetate, 1,140,000 IU; Vitamin D3, 180,000 IU; DL-*α*-tocopherol acetate, 7.6 g; Menadione, 1.2 g; Thiamin nitrate, 0.93 g; Riboflavin, 1.35 g; Pyridoxine hydrochloride, 1.10 g; Cyanocobalamine, 0.0075 g; D-calcium pantothenate, 4.5 g; Nicotinamide, 6.75 g; Folic acid, 0.465 g; D, biotin; 0.0475 g; Inositol, 10 g.

^2^Mineral premix (g/kg premix): FeSO_4_·H_2_O, 112.7 g; ZnSO_4_·H_2_O, 45.2 g; MnSO_4_·H_2_O, 9.3 g; CuSO_4_·5H_2_O, 3.7 g; CoCl_2_·6H_2_O, 0.4 g; Na_2_SeO_3_, 0.1 g; Ca (IO_3_)_2_, 0.3 g.

^3^The DHA-, EPA-, and ARA-enriched oils are purchased from Xi'an Shouhe Biotechnology Co., Ltd. The DHA-enriched oil contained 52% DHA and 9% EPA. The EPA-enriched oil contained 70% EPA and 5% DHA. The ARA-enriched oil contained 48% ARA.

**Table 2:** Fatty acid composition of diets for Chinese tongue sole (% total fatty acids).

Fatty acid	C	DHA-L	DHA-H
14:0	1.36	1.38	1.65
16:0	12.79	12.17	12.05
18:0	3.27	2.95	2.96
20:0	0.16	0.15	0.18
SFA	17.58	16.66	16.83
16:1n-7	1.47	1.31	1.80
17:1n-7	0.16	0.15	0.20
18:1n-9c	31.12	26.66	16.47
20:1n-9	2.75	2.93	2.90
MUFA	35.50	31.06	21.37
18:2n-6c	25.50	25.50	25.11
18:3n-6	0.36	0.42	0.56
20:2n-6	0.89	0.92	0.91
20:4n-6	3.30	3.58	3.40
n-6PUFA	30.05	30.42	29.99
20:5n-3	8.84	9.35	9.45
22:5n-3	0.56	0.86	1.51
22:6n-3	5.16	8.93	16.47
n-3PUFA	14.56	19.14	27.43
n-3/n-6	0.48	0.63	0.91

Abbreviations: C, control; DHA, docosahexaenoic acid; MUFA, monounsaturated fatty acid; PUFA, polyunsaturated fatty acid; SFA, saturated fatty acid.

**Table 3:** Sequences of the primers used in this work.

Primer	(5′−3′) Sequence	GenBank reference	Full-name of a gene
*gnrh2*-F	GGAATCTGAACTGGAGAACTGCT	XM_008321014.3	Gonadotropin-releasing hormone 2
*gnrh2*-R	TGGCTGCTCACAACTTTATCAC		

*gnrh2r*-F	GGAAGAAGAACAGAACGATGCT	XM_17032390.2	Gonadotropin-releasing hormone 2 receptor
*gnrh2r*-R	TGGGTGAAGTTGGCTGGATA		

*gnrh3*-F	AGGCAGCAGAGTGATCGTG	JQ028869	Gonadotropin-releasing hormone 3
*gnrh3*-R	CACCTGGTAGCCATCCATAAGAC		

*fshβ*-F	TGATGGGTGTCCAGAGGAAG	JQ277933	Follicle-stimulating hormone beta
*fshβ*-R	CAACAAACCGTCCACAGTCC		

*lhβ*-F	TCCACCTGACACTAACGCTG	JQ277934	Luteinizing hormone subunit beta
*lhβ*-R	GTTTGGTTCCTTTGTTCTGC		

*pkcα*-F	CGGGTAAGAAGGTAAATGACG	XM_008317323.3	Protein kinase C alpha
*pkcα*-R	GCACTGCCAAAAAGGAGAAG		

*pkcβ*-F	CGACTTCATCTGGGGGTTT	XM_008330122.3	Protein kinase C beta
*pkcβ*-R	AAGGTTGGGCTGGAGTAGGT		

*arrb2*-F	ACGCCCACCCTTTCTACTTC	XM_008333381.3	Arrestin beta 2
*arrb2*-R	TTCTCTTCTACAGACTTGGCACAG		

*mapk*-F	GCTGAGGTGCCATTCAAGTT	XM_008336223.3	Mitogen-activated protein kinase
*mapk-R*	GATAGACGTTAAGACCTAAAGCCA		

*pi3kcα*-F	GGAGTGCGCAAAGACCAAAG	XM_008334702.3	Phosphatidylinositol-4,5-bisphosphate 3-kinase catalytic subunit alpha
*pi3kcα*-R	TCCGGATGTAGGCAATGTCG		

*creb2*-F	CTCCGATACACGAGATTTGACA	XM_008315281.3	cAMP responsive element binding protein-like 2
*creb2*-R	CATTTATTTCTCGGTCTCCCTAC		

*creb31*-F	ATACCCAACAAACTCCCACTCA	XM_008324705.3	cAMP responsive element binding protein-like 3
*creb31*-R	CCAGACATTCCACGTACTCCTT		

*tlr3*-F	GACATTTGAAGAGCCACGACG	XM_008317814.3	Toll-like receptor 3
*tlr3*-R	ACGGGAACCATTGCTGTGATA		

*akt1*-F	CTACAAAGAGCGACCACAAGATG	XM_008323063.2	AKT serine/threonine kinase
*akt1*-R	GCAGGCAGCGGATGATAAA		

*nf-κb1*-F	TGGGAGGCTCTTGGTGATTT	XM_025063059.1	Nuclear factor kappa B subunit 1
*nf-κb1*-R	GGTTTGGGTTCGCTGGTTT		

*nf-κb2*-F	TGGACATCAAGACTGAACCCTAC	XM_025061015.1	Nuclear factor kappa B subunit 2
*nf-κb2*-R	TCACACTCATAACGGAAACGAA		

*fshr*-F	AAGATCAAGGGAAAACGCTA	EU_661784.2	Follicle stimulating hormone receptor
*fshr*-R	CTCAGATGGTTGGAGGAAAG		

*star*-F	ACCTCGTGGGTGACCATCGTGT	NM_001294220.1	Steroidogenic acute regulatory protein
*star*-R	AGGACGGCTGGACCACTGAAAT		

*p450scc*-F	TTCTGTGCTGTATGGCGAAC	XM_008312076.3	Cholesterol side-chain cleavage enzyme
*p450scc*-R	CTTTTGACCCAATCCGTCTC		

*p450c17*-F	GCCCACTCGCTCCCTACATACT	XM_025060511.1	Steroid 17-alpha-hydroxylase/17,20 lyase
*p450c17*-R	GTCTTTCCCATCTCGGGTCAG		

*hsd3b1*-F	CACCACTGGGTAAGCACTATC	XM_008328505.1	Hydroxy-delta-5-steroid dehydrogenase, 3 beta- and steroid delta-isomerase 1
*hsd3b1-R*	AGGTTATCGCAAACAGCATT		

*hsd17b1*-F	AATGTGCAGGCTCTAACTGCTTC	XM_008330027.1	Hydroxysteroid (17-beta) dehydrogenase 1
*hsd17b1*-R	AGGTTCCTCATGGTGGCGTA		

*hsd17b2*-F	GGGCTCTAGTGAACAACGCA	XM_025056010.1	Hydroxysteroid 17-beta dehydrogenase 2
*hsd17b2*-R	AACGGCAGGAAACTCTGACA		

*hsd17b3*-F	CTGCGGAATGAAACTGCTGC	XM_017030963.2	Hydroxysteroid 17-beta dehydrogenase 3
*hsd17b2*-R	GTAACCACTGCCCACTCTCC		

*hsd11b2*-F	TCGTCCATCTTCCAGGTTGC	XM_008310169.3	Hydroxysteroid 11-beta dehydrogenase 2
*hsd11b2*-R	AGAACATCCAGATGCCTCGC		

*p45011b*-F	GTTGGAATCACAGTGCAGCG	XM_025061509.1	Cytochrome P450 11B
*p45011b*-R	CCCAGTGGATACAGACAGGC		

*aromatase*-F	TGCGATTTCAGCCCGT	XM_008310276.3	Cytochrome P450 aromatase
*aromatase*-R	TGCGACCCGTGTTCAGA		

*ar*-F	GGAGAAAGAACTGCCCGTCA	XM_008332997.2	Androgen receptor
*ar*-R	CAGGCTCGCTGTAGGTGAAA		

*β2m*-F	ACGTCCTCGTCTCTTCTGTGAT	FJ965563.1	Beta-2 microglobulin
*β2m*-R	CCAACCTTCTGTTCGCATCT		

*β-actin*-F	GGCTACTCTTTCACCACCACA	XM_008330221.2	Beta-actin
*β-actin*-R	GCCACAGGACTCCATACCAA		

**Table 4:** Antibody information used in this work.

Antibody name	Antibody brand	Product source and code
P450C17	Proteintech	14447-1-AP
HSD17B1	Proteintech	25334-1-AP
*β*-Actin	Proteintech	20536-1-AP

**Table 5:** Sex distribution (number) of the Chinese tongue sole in this experiment.

Sex	C	DHA-L	DHA-H
Genetic females	10	10	7
Male	25	28	25

*Note:* The fish numbers are the sum of the three replicate tanks. Due to mortality in the experiment process, the sum of fish number in each group was less than 45.

Abbreviations: C, control; DHA, docosahexaenoic acid.

**Table 6:** Liver and muscle fatty acid compositions in male Chinese tongue sole (% total fatty acids).

Fatty acid	Liver			Muscle		
	C	DHA-L	DHA-H	C	DHA-M	DHA-H
14:0	1.01 ± 0.13	1.12 ± 0.08	1.14 ± 0.11	1.56 ± 0.10	1.46 ± 0.11	1.25 ± 0.26
16:0	16.5 ± 1.06	16.8 ± 0.67	17.8 ± 0.36	16.4 ± 0.47	16.6 ± 0.09	17.3 ± 1.27
18:0	13.6 ± 1.00	12.2 ± 0.78	12.9 ± 0.55	4.72 ± 0.11	5.32 ± 0.25	5.76 ± 0.81
SFA	31.2 ± 1.93	30.1 ± 1.07	31.9 ± 0.83	22.6 ± 0.46	23.4 ± 0.05	24.3 ± 0.20
14:1n-5	0.31 ± 0.08	0.29 ± 0.06	0.28 ± 0.02	/	/	/
16:1n-7	0.44 ± 0.09	1.16 ± 0.33	0.30 ± 0.06	1.81 ± 0.15	1.70 ± 0.25	1.49 ± 0.10
18:1n-9	15.2 ± 0.86^a^	16.9 ± 0.92^a^	11.5 ± 0.25^b^	23.9 ± 0.93^a^	21.0 ± 0.13^a^	16.2 ± 0.19^b^
MUFA	16.9 ± 1.02^a^	18.9 ± 1.11^a^	12.8 ± 0.18^b^	26.6 ± 0.65^a^	23.9 ± 0.18^b^	18.2 ± 0.09^c^
18:2n-6	16.4 ± 0.19	13.2 ± 1.18	13.9 ± 0.73	18.3 ± 0.39	16.8 ± 0.25	16.8 ± 1.94
20:4n-6	6.00 ± 0.35	4.03 ± 0.63	5.95 ± 0.37	2.94 ± 0.13	2.75 ± 0.08	2.27 ± 0.27
n-6PUFA	22.4 ± 0.44^a^	17.2 ± 1.76^b^	19.9 ± 0.91^ab^	21.2 ± 0.26	19.5 ± 0.17	19.0 ± 1.66
20:5n-3	5.09 ± 0.27	4.09 ± 0.40	4.77 ± 0.20	5.12 ± 0.34	4.90 ± 0.29	2.64 ± 1.93
22:5n-3	1.33 ± 0.19	1.23 ± 0.07	1.68 ± 0.12	2.34 ± 0.12	2.28 ± 0.07	2.32 ± 0.31
22:6n-3	8.22 ± 0.23^a^	9.61 ± 0.41^a^	17.4 ± 0.4^b^	7.01 ± 0.51^a^	9.89 ± 0.00^b^	15.5 ± 0.01^c^
n-3PUFA	14.6 ± 0.67^a^	14.9 ± 0.76^a^	23.8 ± 0.11^b^	14.4 ± 0.29	17.0 ± 0.22	20.4 ± 2.25
n-3/*Σ*n-6	0.65 ± 0.02^a^	0.87 ± 0.04^b^	1.20 ± 0.06^c^	0.68 ± 0.02	0.87 ± 0.00	1.09 ± 0.21

*Note:* For liver or muscle, data in the same row not sharing a superscript were significantly (*p* < 0.05) different. n-6 PUFA, n-6 poly-unsaturated fatty acids; n-3 PUFA, n-3 poly-unsaturated fatty acid. “/” indicates that the fatty acid was not detected in the tissue.

Abbreviations: C, control; DHA, docosahexaenoic acid; MUFA, mono-unsaturated fatty acids; SFA, saturated fatty acids.

## Data Availability

The data that support the findings of this study are available from the corresponding author upon reasonable request.

## Nomenclature

DHA: Docosahexaenoic acid
EPA: Eicosapentaenoic acid
ARA: Arachidonic acid
TFA: Total fatty acid
SFA: Saturated fatty acid
MUFA: Monounsaturated fatty acid
PUFA: Polyunsaturated fatty acid
LC-PUFA: Long-chain polyunsaturated fatty acids
T: Testosterone
11-KT: 11-Ketotestosterone
17*β*-hsd: 17*β*-Hydroxysteroid dehydrogenase
LH: Luteinizing hormone
FSH: Follicle-stimulating hormone
E2: Estradiol.
